# Predictors of objectively measured physical activity in 12‐month‐old infants: A study of linked birth cohort data with electronic health records

**DOI:** 10.1111/ijpo.12512

**Published:** 2019-02-06

**Authors:** Haider Raza, Shang‐Ming Zhou, Charlotte Todd, Danielle Christian, Emily Marchant, Kelly Morgan, Ashrafunnesa Khanom, Rebecca Hill, Ronan A. Lyons, Sinead Brophy

**Affiliations:** ^1^ The School of Computer Science and Electronic Engineering University of Essex Colchester UK; ^2^ Health Data Research UK Swansea University Swansea UK; ^3^ Department of Sport and Physical Activity Edge Hill University Ormskirk UK; ^4^ DECIPHer, School of Social Sciences Cardiff University Cardiff UK; ^5^ Abertawe Bro Morgannwg University Health Board (ABM UHB) Port Talbot UK

**Keywords:** gestation, infants, physical activity, postnatal development

## Abstract

**Background:**

Physical activity (PA) levels are associated with long‐term health, and levels of PA when young are predictive of adult activity levels.

**Objectives:**

This study examines factors associated with PA levels in 12‐month infants.

**Method:**

One hundred forty‐one mother‐infant pairs were recruited via a longitudinal birth cohort study (April 2010 to March 2013). The PA level was collected using accelerometers and linked to postnatal notes and electronic medical records via the Secure Anonymised Information Linkage databank. Univariable and multivariable linear regressions were used to examine the factors associated with PA levels.

**Results:**

Using univariable analysis, higher PA was associated with the following (*P* value less than 0.05): being male, larger infant size, healthy maternal blood pressure levels, full‐term gestation period, higher consumption of vegetables (infant), lower consumption of juice (infant), low consumption of adult crisps (infant), longer breastfeeding duration, and more movement during sleep (infant) but fewer night wakings. Combined into a multivariable regression model (*R* ^2^ = 0.654), all factors remained significant, showing lower PA levels were associated with female gender, smaller infant, preterm birth, higher maternal blood pressure, low vegetable consumption, high crisp consumption, and less night movement.

**Conclusion:**

The PA levels of infants were strongly associated with both gestational and postnatal environmental factors. Healthy behaviours appear to cluster, and a healthy diet was associated with a more active infant. Boys were substantially more active than girls, even at age 12 months. These findings can help inform interventions to promote healthier lives for infants and to understand the determinants of their PA levels.

## 1 INTRODUCTION

Childhood is one of the most important time periods for the formation of future health behaviours.^1^ Evidence shows that the risk factors for many chronic diseases are developed during the early years of life, including the period of pregnancy.^2^ Physical activity (PA) plays an important role in maintaining good health‐related behaviours in children.^3^, ^4^ PA in the early years of life is important for the development of good bone health and advancement of fundamental motor skills.^5^ Communities can help make PA an easy and fun option for the youth.^6^

The early years are a significant period of growth and development, and for this reason, the Department of Health (DH) in the United Kingdom produced guidelines regarding PA for children from birth to 5 years of age.^7^ These guidelines encourage PA from birth, through both floor‐ and water‐based activities. However, little is known about how much activity is normal for infants (birth age, 1 y) and toddlers (1‐3 y). This was highlighted by a recent scoping review,^2^ which found only six studies focusing on measuring and reporting PA levels and patterns in the 0‐ to 2‐year‐old age group. This review noted that most studies in this field had used subjective questionnaires and made reference to the need for employing accelerometery as the main objective tool to measure PA in the early years. Accelerometery does help to capture the intermittent, sporadic nature of PA in this age group^8^ and is not susceptible to reporting bias of parents/carers in questionnaires.

In addition, little is known about the factors that predict PA levels in the very early years. Although many studies have identified predictors of children's PA levels,^9^, ^10^ their focus has been on those older than 2 years of age. Indeed, a recent systematic review of factors associated with PA in children aged 0 to 6 years commented on the lack of high‐quality studies exploring predictive factors and advocated for more longitudinal research in this field.^9^ One study examining factors associated with PA in 2‐year‐old infants found that gender, age, number of siblings, and season of measurement were significant predictors of PA levels. Another longitudinal study found significant positive associations for time spent with other babies of a similar age at 4 months and time spent being active with the child's mother at 9 months.^11^ This suggests that playing with others (other infants, siblings, and parents) may be important for developing good PA levels. However, authors noted that these associations were weak and advocated for further research.

Indeed, comparatively speaking, infancy is the age group for which we understand the least about determinants of PA. What is more, each age group of young children shows quite different stages of motor development and PA patterns,^12^ and evidence about PA in older children cannot be immediately applicable to infants.^8^, ^13^ Infancy could be considered as a critical formative phase for establishing PA. Furthermore, given the emerging evidence for linking adverse health outcomes of young adults to PA levels during childhood, intervention as early as possible in childhood becomes crucial.^1^, ^14^ This may bring greater health benefits than attempting to modify well‐established health‐related behaviours later in adulthood. Enhancing knowledge of the determinants of PA in this group will improve our understanding of when specific patterns of PA and sedentary behaviour develop and inform the earliest possible intervention strategies to promote sustainable, healthy levels of PA through infancy, childhood, and beyond.

To fill this knowledge gap, in this study, factors associated with the PA levels of 12‐month‐old infants were explored, specifically to examine factors that were predictive of higher levels of PA at age 12 months.

## 2 METHODS

### 2.1 Study design, setting, and participants

This study was based on the data collected through the project “The Growing Up in Wales, Environments for Healthy Living (EHL),” which was undertaken during the period of April 2010 to March 2013 in Wales, UK.^15^ The EHL study was designed to complement other epidemiology cohort studies around the world,^15^ adopting a mixed‐methods approach with the use of accelerometery, interviews, questionnaires, and linkage to routinely collected health and education data.

Pregnant women aged 16 years or older, receiving antenatal care within the Abertawe Bro Morgannwg University Health Board (ABM UHB) area south of Wales, UK, were recruited face‐to‐face by researchers at antenatal appointments. After providing informed written consent, women participated in a home visit during pregnancy and a follow‐up visit when the infant was 12 months old. This enabled anthropometric, demographic, and questionnaire data to be collected, including family characteristics, dietary intake, PA, and family‐related variables. Anthropometric data included parental height, weight, four skinfold thicknesses (biceps, triceps, subscapular, and suprailiac), and midarm circumference. Moreover, consent was requested for researchers to access antenatal records, postnatal notes, and routinely collected electronic medical records. Postnatal information was also collected from both participant maternity notes and the parent‐held child health records at the 12‐month follow‐up (including infant weight, length, and infant feeding method); 815 pregnant women were recruited during the period of April 2010 to March 2013, with 422 followed‐up to 12 months. Details of how the women were recruited, when the pregnancy visits were conducted, how data were recorded, etc can be found in the EHL study protocol.^15^

### 2.2 Accelerometer: A triaxial GENEActiv

The current analysis included a total of 141 infants from the EHL study, whose accelerometer‐based readings, alongside questionnaire data (completed by their mothers), and their mother's 12‐month postnatal notes were readily available. A triaxial GENEActiv accelerometer was used to record PA for seven consecutive days for each recruited mother and infant. This device was mounted underneath clothes with good skin contacts on the wrist (mothers) or ankle (infants^16^, ^17^) to collect the movements for each second at 100‐Hz frequency. Given the fact that accelerometers collect data continuously, even when they are not worn, readings were classified into wear and nonwear time events using an existing validated method.^16^ The intensity of activity is measured by the gravity‐subtracted sum of signal vector magnitudes (SVMs). The readings from the accelerometer are signals values in x‐, y‐, and z‐axis, which were summed according to Equation 1.^18^

(1) SVMsg=∑x2+y2+z212−1g.

For each recording in the epoch, the vector magnitude was calculated using Equation 1, and 1*g* (gravitational pull) was subtracted. For example, when the device mounted on the ankle is stationary and the earth's gravitational pull is the only acceleration, the result of this will be zero. The signal was denoted as the movement intensity (MI). The unit for MI is *g* (1*g* = 9.81 m·s^−2^). Statistical measurements for SVMs such as sum, mean, standard deviation, and confidence intervals were used to measure the variations in PA of infants. On the basis of the median of the sum of SVMs over 7 days, the population was divided into two groups (ie, lower and higher PA levels) using the median of the SVMs (n = 141), where the infants with SVM over 4.9754 were regarded as having higher PA levels (see Figure S1 for distribution of PA accelerometry data).

### 2.3 Data linkage and electronic follow‐up

In order to examine the relationship between PA levels and health‐related factors, accelerometer data was linked to the National Community Child Health Database (NCCHD), General Practice (GP) records, and health outcomes via a national privacy protecting e‐health infrastructure, the SAIL databank.^19^ The SAIL databank provides access to a wide range of patient data across different large datasets. The NCCHD provided childhood health and maternal health status data. Therefore, in this study, childhood health, maternal health, and paper‐based postnatal notes were brought together with electronic health records to predict the PA levels of infants. All data are deidentified by an external trusted third party prior to linkage using unique nondisclosable numbers. In this study, birth data regarding the infant were gathered from the NCCHD, and data concerning the delivery of infants and the mother's health status were gathered from GP records. Each linked record had been assigned a match probability. To reduce the bias of data linkage, only the linked records with match probability over 0.90 were used.

### 2.4 Statistical methods

Differences in demographic characteristics and clinical outcomes between the two subgroups (both lower and higher PA levels) are expressed in Table 1. In this selected cohort, some variables presented with missing values (see Figure S2 for distribution of missing values across the studied variables). The *k*‐nearest neighbour (*k*NN) method was used to impute the missing values with 5 neighbours (ie, *k* = 5).^20^ The *k*NN method is an efficient imputation technique by which each missing value on some data fields is replaced with a value calculated from related neighbouring cases across the whole records of a dataset. It has demonstrated good capacity for preserving the original data structure.^20^ For each continuous univariable variable, a two‐sample *t* test was used in between two subgroups at 5% significance level, and a Pearson chi‐squared test was used in categorical variables with 5% significance level. A multiple linear regression model with the outcome variable of PA level was fitted on a set of univariable independent variables to examine whether they are *statistically significant* (*P* values less than 0.05) in distinguishing lower and higher level PA groups. Then all the statistically significant variables were further inputted into a multivariable model to assess their joint relationship with PA level.

**Table 1:** Demographic information of the cohort in this study

Characteristic		Mean ± Std (Lower PA) (n = 70)	Mean ± Std (Higher PA) (n = 71)
Number of	Boys	28	49
	Girls	42	22
Physical activity SVM	Boys	[3.16 ± 1.24]	[6.72 ± 1.32]
	Girls	[3.14 ± 1.17]	[6.63 ± 1.44]
Infant body composition at 12‐mo visit	Length, cm	[62.97 ± 24.36]	84.40 ± 23.40]
	Weight, kg	[9.62 ± 1.82]	[9.65 ± 2.76]
Mother age at 12‐mo visit		[33.57 ± 7.14]	[35.63 ± 6.66]
Mother physical activity SVM		[4.60 ± 2.67]	[5.4 ± 1.24]
Mother body composition at 12‐mo visit	Length, cm	[1.63 ± 0.07]	[1.64 ± 0.08]
	Weight, kg	[68.64 ± 16.31]	[67.89 ± 14.45]
	BMI	[26.48 ± 5.38]	[25.49 ± 5.57]

Abbreviations: BMI, body mass index; PA, physical activity; SVM, signal vector magnitude.

#### 2.4.1 Justification of sample size

The sample size was calculated a priori, in accordance with the primary aim of the study, to distinguish lower and higher level PA groups. For a multiple regression model with nine predictors, a sample size of 113 participants was required to achieve a power of 80% to detect an *R* ^2^ greater than or equal to 15% attributable to the multiple variables in distinguishing these infants, at a 5% significance level.

## 3 RESULTS

### 3.1 Univariable analysis

Accelerometer data were collected for the 141 infants (77 boys and 64 girls). Infants in the higher‐level activity group were more likely to be male (ie, 70.42% boys and 29.58% girls), whereas in the lower activity group, they were more likely to be female (ie, 38.57% boys and 61.43% girls). In this study, the average infant birth weight is 3.44 kg with an average gestational period of 274.10 days. Table 1 provided demographic information of this study (Table S1 showed further information of the infants including the average age when wearing accelerometers, crawling, daycare, days of wearing accelerometers, and number of infants without siblings).

Factors associated with PA level (higher vs lower) were analysed and presented in Table 2. The longer the gestational period, the more active the infant (gestational age 286.08 days compared with 261.96 in the lower active group, mean difference: −24.12 d (95% CI, −34.47 to −13.75). Higher activity infants had more night‐time movement (movement while sleeping) with a mean difference of −1.79 SVM (95% CI, −2.32 to −1.25). Infants with high activity levels drank less juice and ate fewer crisps per week (parental report) compared with infants with low activity levels. However, active infants ate more vegetables and were breastfed for a longer duration (mean difference −7.59 wk [95% CI, −12.84 to −2.33]); the mother was more likely to have tried breastfeeding compared with low activity infants (84.85% mothers of active babies started breastfeeding, compared with 77.61% of low active babies).

**Table 2:** List of predictors associated with lower and higher levels PA groups

(a) List of Categorical Predictors Using Pearson's Chi‐squared Test			
Predictors	Chi‐squared	*df*	*P* value
Gender	10.829	1	< 0.001
Mother's current partner is father	0.2005	1	0.6547
Vehicle availability	0.3008	1	0.5834
Try to breastfeed	0.2300	1	0.6311
Assisted first step	0.1713	1	0.6789
Other adults living at home	0.6482	1	0.4207
Sleep location with parent	0.0922	1	0.7614
Sleeping position (on belly)	0.0060	1	0.9343

(b) List of Continuous Predictors Using Two‐sample *t* test				
Predictor	Lower PA (n = 70) Mean ± Std	Higher PA (n = 71) Mean ± Std	Mean Difference	CI
Birth weight, kg	[3.20 ± 0.92]	[3.18 ± 0.79]	0.02	[−0.27 to 0.30]
Head circumference‐birth, cm	[32.93 ± 7.84]	[33.70 ± 9.52]	−0.77	[−3.67 to 2.13]
APGAR‐1 score	[7.53 ± 2.27]	[8.34 ± 2.71]	−0.81	[−1.63 to 0.03]
APGAR‐2 score	[9.92 ± 1.20]	[9.95 ± 1.39]	−0.03	[−0.46 to 0.40]
Head circumference, cm (12 mo)	[43.24 ± 8.59]	[44.66 ± 14.49]	−1.42	[−5.38 to 2.56]
Infant length, cm (12 mo)	[62.97 ± 24.36]	84.40 ± 23.40]	−21.43	[−29.38 to −13.47]
Infant weight, kg (12 mo)	[9.62 ± 1.82]	[9.65 ± 2.76]	−0.03	[−0.80 to 0.75]
Infant biceps, cm (12 mo)	[8.24 ± 3.56]	[9.86 ± 4.05]	−1.62	[−2.89 to −0.34]
Infant triceps, mm (12 mo)	[12.96 ± 8.26]	[13.39 ± 7.87]	−0.43	[−1.90 to 1.53]
Mother weight, kg (12 mo)	[68.64 ± 16.31]	[67.89 ± 14.45]	0.75	[−3.12 to 2.25]
Mother biceps, cm (12 mo)	[12.47 ± 6.96]	[11.18 ± 4.71]	1.29	[−0.69 to 3.26]
Mother triceps, mm (12 mo)	[19.87 ± 13.47]	[22.34 ± 13.27]	−2.47	[−6.91 to 1.99]
Mother systolic BP, mmHg (12 mo)	[103.63 ± 41.04]	[138.68 ± 46.41]	−35.05	[−49.64 to −20.44]
Mother diastolic BP, mmHg (12 mo)	[75.29 ± 36.97]	[91.91 ± 29.15]	16.62	[−27.70 to −5.54]
Gestation period, d	[261.96 ± 40.18]	[286.08 ± 18.14]	−24.12	[−34.47 to −13.75]
Fruits PW	[13.66 ± 8.81]	[11.88 ± 7.09]	1.78	[−0.88 to 4.43]
Vegetable PW	[9.04 ± 6.34]	[11.36 ± 6.40]	−2.32	[−4.44 to −0.19]
Meat PW	[5.93 ± 2.57]	[5.85 ± 2.78]	0.08	[−0.81 to 0.97]
Fish PW	[2.00 ± 1.90]	[4.14 ± 10.51]	−2.14	[−4.66 to 0.38]
Juice PW	[1.73 ± 2.94]	[0.87 ± 1.81]	0.86	[0.04 to 1.67]
Water PW	[5.59 ± 5.67]	[6.63 ± 8.20]	−1.04	[−3.39 to 1.31]
Tea PW	[0.91 ± 2.45]	[0.98 ± 2.59]	−0.07	[−0.90 to 0.77]
Squash PW	[1.51 ± 2.88]	[1.92 ± 3.12]	−0.41	[−1.40 to 0.59]
Cheese PW	[1.8 ± 1.85]	[2.09 ± 1.73]	−0.29	[−0.89 to 0.29]
Yogurt PW	[3.70 ± 2.29]	[3.36 ± 2.12]	0.34	[−0.392 to 1.08]
Chocolate PW	[0.57 ± 0.89]	[0.56 ± 0.98]	0.01	[−0.29 to 0.32]
Adult crisps PW	[0.72 ± 1.10]	[0.33 ± 0.93]	0.39	[0.05 to 0.73]
Snack biscuits PW	[2.22 ± 2.39]	[2.42 ± 2.08]	0.20	[−0.94 to 0.55]
# meals home‐made PW	[11.74 ± 8.07]	[14.18 ± 8.23]	−2.44	[−5.15 to 0.27]
# jar box PW	[5.42 ± 5.21]	[6.42 ± 5.26]	−1	[−2.73 to 0.75]
# meal eaten out PW	[4.19 ± 3.84]	[5.07 ± 3.59]	−0.88	[−2.12 to 0.36]
# meal same as mom PW	[4.71 ± 4.02]	[5.34 ± 6.82]	−0.63	[−2.49 to 1.24]
# meal same as dad PW	[4.48 ± 4.11]	[5.00 ± 6.70]	−0.52	[−2.37 to 1.33]
# Milk feed PW	[9.48 ± 12.68]	[7.91 ± 10.61]	1.57	[−2.31 to 5.47]
Breastfeeding duration, wk	[16.44 ± 13.57]	[24.03 ± 17.70]	−7.59	[−12.84 to −2.33]
Age first formula Milk, wk	[7.31 ± 9.87]	[9.45 ± 11.68]	−2.14	[−5.74 to 1.46]
Mother age, y	[32.95 ± 8.55]	[33.55 ± 9.97]	−0.6	[−3.69 to 2.49]
Sleeping hours (night)	[10.21 ± 2.13]	[10.52 ± 1.63]	−0.31	[−0.93 to 0.32]
Sleeping hours (day)	[1.96 ± 1.23]	[2.10 ± 0.92]	−0.14	[−0.49 to 0.23]
# night wakes	[1.41 ± 1.63]	[1.04 ± 1.26]	0.37	[−0.11 to 0.86]
Sleepless hours in night	[1.47 ± 3.37]	[1.10 ± 2.51]	0.37	[−0.61 to 1.36]
Time taken to put sleep, min	[31.59 ± 71.89]	[33.96 ± 81.91]	−2.37	[−28.04 to 23.30]
Infant's time of sleep	[18.90 ± 3.76]	[18.64 ± 3.10]	0.26	[−0.88 to 1.40]
Age‐sleep through night, m	[4.68 ± 2.99]	[6.49 ± 3.43]	−1.81	[−4.37 to 0.75]
Crawl age, m	[9.22 ± 5.15]	[8.98 ± 4.66]	0.24	[−1.39 to 1.87]
Movements during sleep time	[1.53 ± 0.66]	[3.32 ± 2.16]	−1.79	[−2.32 to −1.25]
Number of sport activity or PA in day	[1.35 ± 1.09]	[1.13 ± 0.47]	0.22	[−0.05 to 0.50]
Number of normal activity in day	[0.95 ± 0.13]	[1.02 ± 0.31]	−0.07	[−0.14 to 0.01]
Maternity leave, m	[4.99 ± 1.47]	[5.07 ± 1.31]	−0.08	[−0.54 to 0.38]
Income, £	[50476 ± 26239]	[52462 ± 21940]	−1986	[−10435 to 6464.6]

Abbreviations: *df*, degree of freedom; PA, physical activity.

In addition, active infants were larger in terms of longer length (mean difference −21.43 cm [95% CI, −29.38 to −13.47]) and had thicker biceps (mean difference −1.62 cm [95% CI, −2.89 to −0.34]).

Factors not associated with PA level in infants included birth weight, head circumference, APGAR‐1 or APGAR‐2 score, maternal age, maternal weight, maternity leave duration, time taken to put asleep and time to sleep, presence of other members at home, co‐sleeping with a parent, sleeping position, maternal everyday sports, and maternal income. Active infants tended to eat more homemade meals, but this finding was not statistically significant (*P* value = 0.077; CI, −5.15 to 0.27). Active infants also had better sleeping habits, ie, less night awakenings (*P* value = 0.130; CI, −0.11 to 0.86).

### 3.2 Multivariable analysis

All variables used in the univariable analysis are presented in Table 2a (categorical variables) and Table 2b (continuous variables). Among all continuous and categorical variables, only significant variables (*P* values less than 0.05) were analysed in the multivariable model for the 141 infants, with the variable gender as a categorical variable and all other variables as continuous variables.

To find the best solution among all possible options in fitting the linear regression model, the *R* ^2^ value–guided model selection scheme was used to identify the best model with a maximum *R* ^2^ value (ie, a set of predictors that generate a maximum *R* ^2^ value was selected). Different statistical tests were conducted to validate the assumptions underlying the regression.^21^, ^22^ First tests for multicollinearity were performed, and no variables were found to be collinear (see Figure S3 for the correlation coefficients. Due to collinearity of systolic blood pressure with diastolic blood pressure, only the diastolic blood pressure variable was included). The Kolmogorov‐Smirnov test was conducted to validate the normality of the residuals of the regression (*P* = 0.939) (see Figure S4). Then the Durbin‐Watson test validated that the residuals from the regression were uncorrelated with the statistic value 2.0628. Finally, we validated that the data were homoscedastic, where the residuals of regression were equally distributed (see Figure S5).

**Figure 1:**
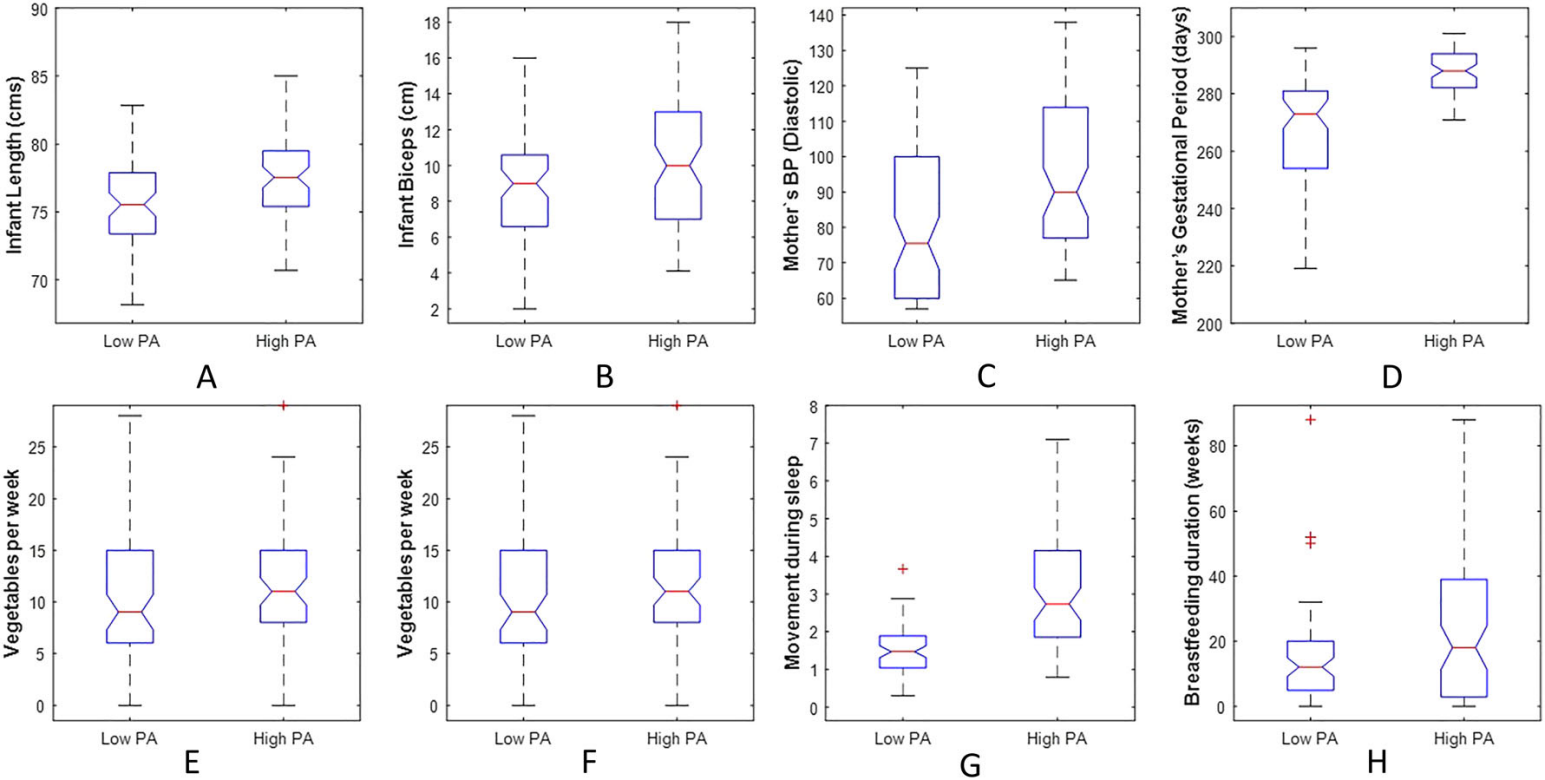
Boxplots for the independent variables, the whiskers describe the full extent of the data; the box corresponds to the lower quartile, median, and upper quartile

The model with the best *R* ^2^ value (*R* ^2^ = 0.654) is presented in Table 3. In this model, higher PA level of infants aged 12 months is associated with male gender, larger infant (infant length, infant biceps size), maternal normal diastolic blood pressure, longer gestational age, higher vegetable consumption per week (infant), lower crisp intake per week (infant), and higher movement during sleep time (infant). A boxplot is illustrated in Figure 1, where each variable in (A) to (H) was an independent variable identified by multivariable regression model.

**Table 3:** Linear regression model with grid search method

Predictors	Estimate	SE	*t* stat	*P* value	CI
Gender (male)	−0.67815	0.025261	−0.6846	0.0082*	[0.0125 to 0.8215]
Infant length	−0.060057	0.031159	−1.9275	0.046086*	[−0.012 to −0.001]
Infant biceps	0.099248	0.044986	2.2062	0.029113*	[0.010 to 0.188]
Mother's diastolic blood pressure	−0.019507	0.0050473	−3.8649	0.00017405*	[−0.029 to −0.009]
Gestational age	0.037241	0.0046645	7.9839	6.3111e‐13*	[0.028 to 0.046]
Vegetable consumed per week	0.037054	0.018818	1.9691	0.048054*	[0.001 to 0.074]
Adult crisp consumed per week	−0.27719	0.099358	−2.7898	0.006062*	[−0.473 to −0.080]
Movement during sleep time	0.5843	0.071699	8.1494	2.5533e‐13*	[0.442 to 0.726]
Age of last breastfed	0.01073	0.0066951	1.6027	0.11141	[−0.002 to 0.023]

* *P* values <0.05.

## 4 DISCUSSION

This study is, to our knowledge, the first of its kind to examine factors associated with objectively measured activity levels in 12‐month‐old infants, combined with objective assessments of a wide range of explanatory variables obtained through home visits and use of routine electronic health records.

Existing evidence suggests that at age 5 to 6 years, boys are more active than girls.^23^ This study has shown that boys were more active than girls as early as 12 months old. This may raise important questions regarding PA for girls. For example, are boys inherently more physically active than girls? Or are girls treated differently and less encouraged to be active compared with boys? A previous study in children aged 2 to 5 years showed boys to be significantly more active than girls.^24^ Another study looked into children aged 19 months and highlighted that boys engaged in more moderate to vigorous PA than girls,^25^ displaying significantly higher PA during morning hours. Our study finds the same gender differences as early as 12 months. This finding does merit further research to examine if measures to increase girls to be active are hindered by lower natural inherent activity levels in girls, or if gender stereotypes and how parents engage with their children differs by gender.

We have further identified a number of potentially modifiable factors associated with higher activity levels in infants, such as longer breastfeeding duration, lower consumption of juice, and higher consumption of vegetables, which suggests either that diet is important in PA or that healthy behaviours cluster in the same families/individuals.

Regular exercise can bring health benefits for reducing high blood pressure in adults and preschool children,^26^, ^27^, ^28^, ^29^ considering hypertension is strongly related to cardiovascular disease and all‐cause mortality. Our study generated new evidence that normal maternal blood pressure is positively associated with higher PA level of infants aged 12 months. This may suggest that the mothers of active infants are more active themselves.

It is not surprising that a longer gestational period is associated with higher activity levels given the motor development implications of preterm births^30^ and the gradient of worsening health outcomes with decreasing gestational length, including for general health status (hospital admissions and longstanding illnesses), growth/weight gain, and asthma/wheeze.^31^ This implies that measures to prevent preterm birth could have additional benefits in improving activity levels in infants. Therefore, our finding that full‐term infants are more active fits with a growing literature that preterm birth adversely affects future health and well‐being.

Breastfeeding duration has been associated with many positive outcomes with studies showing a protective relationship against being overweight during childhood and adolescence.^32^ In this study, there is evidence of such a protective influence of breastfeeding on infant PA levels, with infants who were breastfed for longer more likely to be in the high activity group. Few studies have explored the influence of breastfeeding duration on PA level. Longer breastfeeding duration has been associated with improved motor development,^33^ higher cardiorespiratory fitness,^34^ and increased explosive lower body strength in children and adolescents.^35^ However, other studies have found no such association with fitness.^36^ Potentially, as above, healthy behaviours cluster in the same individuals, and mothers that breastfeed are also more likely to engage in active play with their infants. This may provide some rationale for the association between lower adult crisp and juice intake and PA, showing health beliefs leading to clustering of health behaviours.

However, limitations of this study include the generalizability of study findings, as we only focused on infants wearing accelerometers on their ankles in order to generate consistent PA levels across these infants. In addition, in this study, 7 days duration of wear was selected as it provided a good representation of habitual lifestyles covering both weekdays and weekends. Nevertheless, selecting 7 days may favour highly representative data for those individuals, while this is at the expense of a reduced sample size as all the infants who had less than 7 days wear were excluded from the study. Other potential limitations are that we did not know the age of motor milestones, such as started crawling/walking to adjust for, and the number of siblings of the infants and the length of time for parents playing with the infants, which are certainly topics meriting further research.

## 5 CONCLUSION

This study identified the factors associated with PA levels in 12‐month‐old infants. Strongly significant associations were found between PA levels of infants and both gestational and postnatal environmental factors including gestational age, breastfeeding duration, mother's blood pressure at 12 months, infant and bicep lengths, vegetable, juice, and crisps intake, and infant's movement during sleep time. There was no significant effect on the weight of the baby in terms of activity levels; however, preterm birth was associated with lower activity levels. This study also found that boys were substantially more active than girls at age 12 months and suggests that higher activity level in infants clusters with other healthy behaviours such as eating vegetables, breastfeeding, and good maternal health. This study suggests that improving infant activity level interventions should target general family health such as facilitating family activity and helping to improve family diet.

## CONFLICTS OF INTEREST

The authors declare that they do not have any commercial or associative interest that represents a conflict of interest in connection with the work submitted.

## AUTHOR CONTRIBUTION

S.B. and R.H. conceived the study. S.M.Z. and H.R. conceived data analysis methods. H.R. and S.M.Z. conducted the statistical analysis. S.B., S.M.Z., and H.R. analysed the results. H.Z., S.M.Z., and C.T. wrote up the manuscript. R.H. collected data in the cohort. R.A.L. received funding for EHL, advised on study design, and reviewed the outcomes. C.T., D.C., E.M., K.M., and A.K. collected and validated data for EHL. All authors reviewed and approved the final manuscript.

## ETHICAL APPROVAL

Ethical approval for the Growing Up in Wales, Environments for Healthy Living (EHL) study was granted through the South East Wales Research Ethics Committee (09/WSE02/37). All participant data were securely anonymised and encrypted via the SAIL databank, so that individuals could not be identified by the analysis team.

## Supporting information

Table S1. More demographics of the infants in this study
Figure S1: The PA accelerometry data: (left) Distribution of PA levels; (right) PA levels in ascending order
Figure S2: Distribution of numbers of missing values across the studied variables.
Figure S3: Pearson correlation coefficient heat map. Correlations are scaled by the colour of the corresponding cell and the variables considered for the multivariable linear regression model are represented in the same order on the x‐ and y‐axes. Gender‐male (G‐M), Gender‐female (G‐F), infant length (IFL), infant biceps (IFB), blood pressure diastolic (BPD), blood pressure systolic (BPS), gestation period in days (GDs), vegetable per week (VPW), juice per week (JPW), adult crisp packets per week (CPW), movement during night‐time (MNT), length on breastfeeding in weeks (LBW).
Figure S4: Normal probability plot of the residuals of the fitted model.
Figure S5: Homoscedasticity of the residuals in the regression analysis
