# Supplementary material for: Predictors of objectively measured physical activity in 12‐month‐old infants: A study of linked birth cohort data with electronic health records
Source: Pediatr Obes. 2019 Feb 6;14(7):e12512. doi: 10.1111/ijpo.12512 (PMC6563068; doi:10.1111/ijpo.12512)
Supplement: Supplementary file 1 — Table S1. More demographics of the infants in this study Figure S1: The PA accelerometry data: (left) Distribution of PA levels; (right) PA levels in ascending order Figure S2: Distribution of numbers of missing values across the studied variables. Figure S3: Pearson correlation coefficient heat map. Correlations are scaled by the colour of the corresponding cell and the variables considered for the multivariable linear regression model are represented in the same order on the x‐ and y‐axes. Gender‐male (G‐M), Gender‐female (G‐F), infant length (IFL), infant biceps (IFB), blood pressure diastolic (BPD), blood pressure systolic (BPS), gestation period in days (GDs), vegetable per week (VPW), juice per week (JPW), adult crisp packets per week (CPW), movement during night‐time (MNT), length on breastfeeding in weeks (LBW). Figure S4: Normal probability plot of the residuals of the fitted model. Figure S5: Homoscedasticity of the residuals in the regression analysis [file IJPO-14-na-s001.pdf]

# Supplementary Material

## Predictors of Objectively Measured Physical Activity in 12 month-Old Infants: A Study of Linked Birth Cohort Data with Electronic Health Records

**Haider Raza, PhD[1+], Shang-Ming Zhou, PhD[1*+], Charlotte Todd[1], Danielle Christian, PhD[1], Emily Marchant, PhD Student [1], Kelly Morgan, PhD[2], Ashrafunnesa Khanom, PhD [1], Rebecca Hill, PhD[3], Ronan A Lyons, MD [1], Sinead Brophy, PhD [1+]**

[1]Health Data Research UK, Swansea University, Swansea, UK.
[2]DECIPHer, School of Social Sciences, Cardiff University, UK.
[3]Abertawe Bro Morgannwg University Health Board (ABM UHB), Port Talbot, UK.
[+]Joint lead authors.
[*]*Corresponding author*: s.zhou@swansea.ac.uk

sTable 1. More demographics of the infants in this study

| **Characteristic** | **Value** |
|---|---|
| Average age of infant (in weeks) when wearing accelerometers | 66.98 |
| Number of crawling infants | 44 |
| Number of infants in daycare[*] | 11 |
| Days of infants wearing accelerometers | 6.94 |
| Number of infants without siblings | 36 |

[*]Infants were cared for by registered or unregistered childminder, workplace/college nursery/crèche, local authority nursery/crèche, or private nursery/crèche.

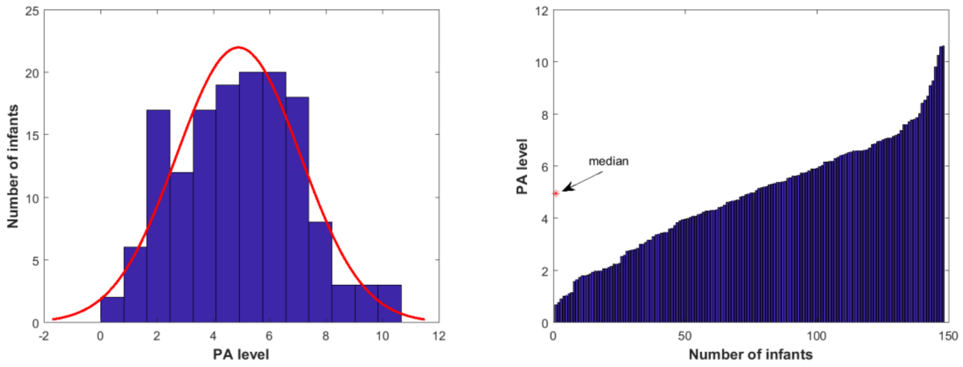


sFigure 1: The PA accelerometry data: (left) Distribution of PA levels; (right) PA levels in ascending order

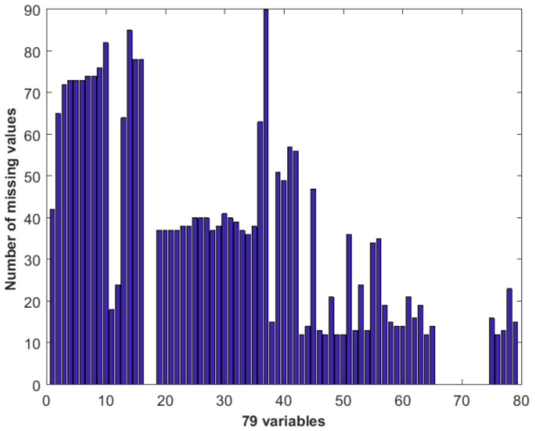


sFigure 2: Distribution of numbers of missing values across the studied variables.

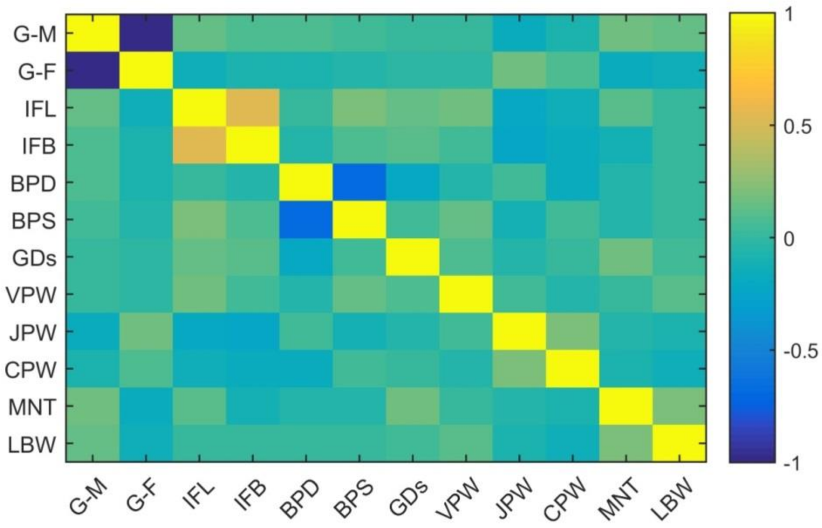


sFigure 3: Pearson correlation coefficient heat map. Correlations are scaled by the colour of the corresponding cell and the variables considered for the multivariable linear regression model are represented in the same order on the x- and y-axes. Gender-male (G-M), Gender-female (G-F), infant length (IFL), infant biceps (IFB), blood pressure diastolic (BPD), blood pressure systolic (BPS), gestation period in days (GDs), vegetable per week (VPW), juice per week (JPW), adult crisp packets per week (CPW), movement during night-time (MNT), length on breastfeeding in weeks (LBW).

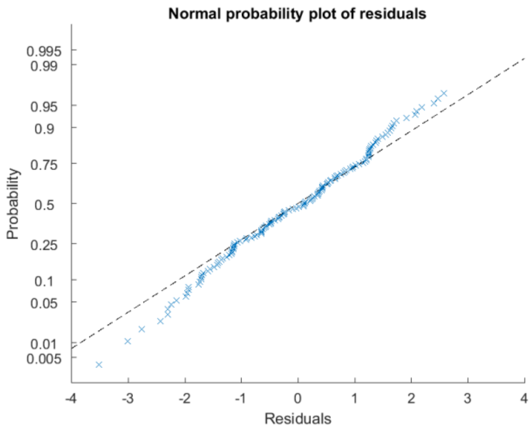


sFigure 4: Normal probability plot of the residuals of the fitted model.

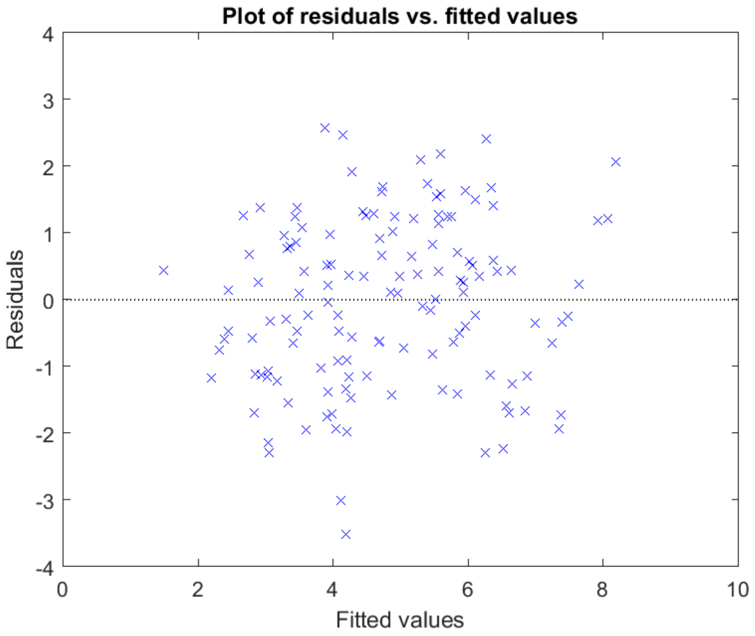


sFigure 5: Homoscedasticity of the residuals in the regression analysis
